# Reactive Oxygen Species-Related Ceftazidime Resistance Is Caused by the Pyruvate Cycle Perturbation and Reverted by Fe^3 +^ in *Edwardsiella tarda*

**DOI:** 10.3389/fmicb.2021.654783

**Published:** 2021-04-28

**Authors:** Jinzhou Ye, Yubin Su, Xuanxian Peng, Hui Li

**Affiliations:** ^1^Center for Proteomics and Metabolomics, State Key Laboratory of Biocontrol, Southern Marine Science and Engineering Guangdong Laboratory (Zhuhai), School of Life Sciences, Sun Yat-sen University, Guangzhou, China; ^2^Key Laboratory of Functional Protein Research of Guangdong Higher Education Institutes, Department of Biotechnology, College of Life Science and Technology, Jinan University, Guangzhou, China; ^3^Laboratory for Marine Fisheries Science and Food Production Processes, Qingdao National Laboratory for Marine Science and Technology, Qingdao, China

**Keywords:** antibiotic resistance, reactive oxygen species, *Edwardsiella tarda*, the pyruvate cycle, ceftazidime

## Abstract

Reactive oxygen species (ROS) are related to antibiotic resistance and have been reported in bacteria. However, whether ROS contribute to ceftazidime resistance and plays a role in ceftazidime-mediated killing is unknown. The present study showed lower ROS production in ceftazidime-resistant *Edwardsiella tarda* (LTB4-R_*CAZ*_) than that in LTB4-sensitive *E. tarda* (LTB4-S), two isogenic *E. tarda* LTB4 strains, which was related to bacterial viability in the presence of ceftazidime. Consistently, ROS promoter Fe^3+^ and inhibitor thiourea elevated and reduced the ceftazidime-mediated killing, respectively. Further investigation indicated that the reduction of ROS is related to inactivation of the pyruvate cycle, which provides sources for ROS biosynthesis, but not superoxide dismutase (SOD) and catalase (CAT), which degrade ROS. Interestingly, Fe^3+^ promoted the P cycle, increased ROS biosynthesis, and thereby promoted ceftazidime-mediated killing. The Fe^3+^-induced potentiation is generalizable to cephalosporins and clinically isolated multidrug-resistant pathogens. These results show that ROS play a role in bacterial resistance and sensitivity to ceftazidime. More importantly, the present study reveals a previously unknown mechanism that Fe^3+^ elevates ROS production via promoting the P cycle.

## Introduction

*Edwardsiella tarda* is known for causing diseases in both humans and fish, in both of which these diseases can potentially be fatal if untreated. In aquaculture, the bacterium targets at a wide range of fish species and thereby leads to extensive economic losses in the industry (Wang et al., 2011; Abayneh et al., 2013). Various antibiotics are used to prevent and control the infections caused by the bacterium. Unfortunately, the overuse of antibiotics has inadvertently promoted the emergence and rapid spread of antibiotic-resistant bacteria (Cabello et al., 2016). The emergence of antibiotic-resistant bacteria poses a major challenge for health practitioners and a huge threat to human health and aquaculture since antibiotic-resistant bacteria are insensitive to antibiotics. As the process of developing new pharmaceutical agents to control antibiotic-resistant pathogens is slow and not a viable approach to manage the growing infectious diseases, further understanding of antibiotic resistance mechanisms for control of these antibiotic-resistant pathogens is an important scientific issue and becomes a major research focus (Defoirdt et al., 2011; Blair et al., 2015).

A line of evidences has indicated that microbial metabolic environment confounds antibiotic sensitivity (Peng et al., 2015; Yao et al., 2016; Cheng et al., 2019; Stokes et al., 2019; Jiang et al., 2020a; Li et al., 2020), where reactive oxygen species (ROS) are related to bacterial resistance to antibiotics and antibiotic-mediated killing efficacy (Dwyer et al., 2009; Van Acker and Coenye, 2017; Zhao and Drlica, 2014). The tricarboxylic acid cycle (TCA) cycle plays a crucial role in ROS formation (Van Acker and Coenye, 2017). Therefore, antibiotic-resistant bacteria exhibit the occurrence of reduced or fluctuated TCA cycle and decreased ROS (Ye et al., 2018; Zhang et al., 2020), indicating that low ROS concentrations induce resistance (Van Acker and Coenye, 2017). In the antibiotic-mediated killing mechanisms, antibiotics belonging to different classes activate the TCA cycle, supporting the formation of ROS (superoxide and hydrogen peroxide) via hyperactivation of the electron transport chain (Dwyer et al., 2009; Van Acker and Coenye, 2017). Fe^3+^ causes the activation of the Fenton reaction to generate abundant ROS against methicillin−resistant *Staphylococcus aureus* (MRSA) infection (Song et al., 2020). Our recent publications have showed that the ROS induced by exogenous metabolites elevate aminoglycoside-mediated killing efficacy to EIB202 and *Vibrio alginolyticus* (Ye et al., 2018; Zhang et al., 2020). Reports also indicate that the beta-lactam stress increased the intracellular ROS level (Rosato et al., 2014; Huang et al., 2019), but whether ROS promote beta-lactam-mediated killing is unknown. Therefore, further understanding of ROS role is required for control of beta-lactam-resistant pathogens.

Ceftazidime is a semisynthetic, broad-spectrum, beta-lactam antibiotic, playing a bactericidal action by inhibiting enzymes responsible for cell wall synthesis, primarily penicillin-binding protein 3 (PBP3). The drug is a commonly used antibiotic in clinics. However, with a wide use of cephalosporins in clinics, resistance to cephalosporins including ceftazidime is predominant. Inactivation of β-lactams by β-lactamases, failure in binding to penicillin-binding proteins, and alteration of binding affinity to penicillin-binding proteins are identified as the three common mechanisms of resistance to β-lactams (Peng et al., 2019). However, whether ROS play a role in ceftazidime sensitivity and resistance is largely unknown. Furthermore, information regarding mechanisms for Fe^3+^-mediated ROS is not available.

In this study, we showed that the intracellular ROS production was lower in LTB4-R_*CAZ*_ than that in LTB4-S due to a decrease of ROS generation. The decrease of ROS generation was attributed to inactivation of the pyruvate cycle (the P cycle) (Ye et al., 2018). Fe^3+^ promoted the P cycle for elevation of ROS production, thereby elevating ceftazidime-mediated killing. These results are described below.

## Materials and Methods

### Bacterial Strains and Culture Conditions

*Edwardsiella tarda* LTB4 used in this study was obtained from Professor Xiaohua Zhang, Ocean University of China University. LTB4 was grown at 30°C for 24 h in 50 ml Luria-Bertani (LB) broth in 250-ml flasks.

### Measurement of Minimum Inhibitory Concentration

Measurement of minimum inhibitory concentration (MIC) was performed as previously described (National Committee for Clinical Laboratory Standards, 1999). In brief, LTB4 was cultured in LB medium with twofold serially diluted ceftazidime (CAZ, Guangzhou QiYun Biological Technology) ranging from 0.01 to 160 μg/ml. Overnight bacterial cultures were diluted 1:100 in fresh LB medium and cultured at 30°C to an OD600 of 0.5. The tray contained a series of twofold dilutions of antibiotics. Ninety microliters of LB containing CAZ and 10 μl of logarithmic phase cells with 10^7^ CFU/ml of LTB4-R_*CAZ*_ or LTB4-R_*CAZ*_ were incubated in a microwell plate for 24 h at 30°C. The lowest concentration showing no visible growth was recorded as the MIC. At least three biologic replicates were performed.

### Real-Time Quantitative PCR

Real-time quantitative PCR (qRT-PCR) was carried out as previously described (Li et al., 2016). Bacterial cells were harvested at OD600 = 1.0. The total RNA of each sample was isolated with TRIzol (Invitrogen, United States). Reverse transcription-PCR was carried out on a PrimeScript^TM^ RT Reagent Kit with gDNA eraser (Takara, Japan) with 1 μg of total RNA according to manufacturer’s instructions. qRT-PCR was performed in 384-well plates, and each well contained a total volume of 10 ml liquid including 5 ml 2X SYBR Premix Ex Taq^TM^, 2.6 μl PCR-grade water, 2 μl cDNA template, and 0.2 μl each pair of primers (10 mM). The primers are listed in Supplementary Table 1. All the assays were performed on the LightCycler 480 system (Roche, Germany) according to the manufacturer’s instructions, and four independent samples were assayed for both the control group and the test group. The cycling parameters were listed as follows: 95°C for 30 s to activate the polymerase; 40 cycles of 95°C for 10 s; and 60°C for 30 s. Fluorescence measurements were performed at 70°C for 1 s during each cycle. Cycling was terminated at 95°C with a calefactive velocity of 5°C per second, and a melting curve was obtained. To analyze the relative expression level of the target gene, we converted the data to percentages relative to the value of no-treatment group. At least triplicate biological repeats were carried out.

### Metabolomics Analysis

Bacterial sample preparation was carried out as previously described (Zhang et al., 2019). In brief, 10 ml OD600 = 1.0 cells were quenched with cold methanol and sonicated for 5 min at 200 W. Samples were centrifuged at 12,000 rpm for 10 min. Supernatant, containing 1 μg/ml ribitol (Sigma–Aldrich) as internal analytical standard, was transferred into a new tube and dried by vacuum centrifugation device (LABCONCO). The dried extracts were then incubated with 80 μl methoxyamine hydrochloride (20 mg/ml, Sigma–Aldrich) in pyridine (Sigma–Aldrich) for 90 min at 37°C and derivatization was done with an identical volume of N-methyl-N-(trimethylsilyl)trifluoroacetamide (Sigma–Aldrich) for another 30 min. Samples were centrifuged at 12,000 rpm for 10 min, and the supernatant was transferred into new tubes. Gas chromatography-mass spectrometry (GC-MS) analysis was performed with an Agilent GC-MS instrument. Spectral deconvolution and calibration were performed using AMDIS and internal standards as previously described. A retention time (RT) correction was performed for all the samples, and then the RT was used as a reference against which the remaining spectra were queried, and a file containing the abundance information for each metabolite in all the samples was assembled. Metabolites from the GC-MS spectra were identified by searching in the National Institute of Standards and Technology (NIST11.L) Mass Spectral Library. Among the detected peaks of all chromatograms, compound peaks were considered as endogenous metabolites and the same metabolite names were merged. The resulting data matrix was normalized by the concentrations of added internal standards and the total intensity. This file was then used for subsequent statistical analyses. The abundance of a metabolite was scaled by total abundance of all metabolites in a sample as its relative abundance for further analysis. Hierarchical clustering was completed in the R platform with the package gplots^1^ using the distance matrix. Multivariate statistical analysis included principal component analysis (SIMCA-P 12.0.1), which was used to discriminate sample patterns. GraphPad Prism 7 was used to draw figures.

### Measurement of the Activity of Enzymes in the P Cycle

Measurement of enzyme activity was performed as previously described with a few modifications (Zhang et al., 2019; Jiang et al., 2020c). In brief, the harvested cells were collected at OD600 = 1.0 and then re-suspended in sterile saline to OD600 = 1.0 after washing. Samples of 30 ml were collected by centrifugation at 8,000 rpm for 5 min. Pellets were re-suspended in phosphate-buffered saline (PBS) and broke down by sonication for 2 min at a 200-W power setting on ice, and then centrifuged at 12,000 rpm for 10 min to remove insoluble materials. Supernatants containing 200 μg of total proteins were transferred to a pyruvate dehydrogenase (PDH) reaction mix (0.15 mM MTT, 1 mM MgCl_2_, 0.5 mM PMS, 0.2 mM TPP, 2 mM sodium pyruvate, and 50 mM PBS), an α-ketoglutarate dehydrogenase (KGDH) reaction mix (0.15 mM MTT, 1 mM MgCl_2_, 0.5 mM PMS, 0.2 mM TPP, 50 mM α-ketoglutaric acid potassium salt, and 50 mM PBS), a succinate dehydrogenase (SDH) reaction mix (0.15 mM MTT, 1 mM PMS, 5 mM sodium succinate, and 50 mM PBS), and a malate dehydrogenase (MDH) reaction mix (0.15 mM MTT, 1 mM PMS, 50 mM PBS, and 50 mM malate) to a final volume of 200 μl in a 96-well plate. Subsequently, the plate was incubated at 37°C for 5 min for PDH/KGDH/SDH/MDH assay and then measured at 566 nm for colorimetric readings. The plate was protected from light during the incubation. The dehydrogenase activity was assayed in quadruplicate.

### Detection of Superoxide Dismutase and Catalase Activity

The activity of superoxide dismutase (SOD) and catalase (CAT) was determined by commercial kits (Nanjing Jiancheng, China). The process is as follows: 10 ml of bacteria with OD600 = 1.0 was collected, washed twice with PBS, and suspended in PBS. The suspension was broke down by sonication for 2 min at a 200-W power setting on ice and then centrifuged at 12,000 rpm for 10 min to remove insoluble materials. The supernatant was taken, and the protein concentration was determined. The activity of SOD was measured with 150 μg total protein. The follow-up test was carried out according to the manufacturer’s instructions. Then, SOD activity was measured at 450 nm, and CAT activity was measured at 405 nm. The plate was protected from light during the incubation.

### Measurement of ATP

Detection of ATP was determined by a BacTiter-Glo^TM^ Microbial Cell Viability Assay (Cat. G8231, Promega, Madison, WI, United States) as previously described (Cheng et al., 2019). In brief, bacterial cells were harvested in the OD600 of 1.0 by centrifugation for 10 min at 12,000 *g* and washed twice by centrifugation with sterile saline. Cells were resuspended with saline solution and adjusted the OD600 to 1.0. Then, 50-μl samples were added to a 96-well plate and mixed with an equal volume of the kit solution. Then, the absorbance was measured using VICTOR X5 (PerkinElmer, Turku, Finland) according to the manufacturer’s instructions. The concentration of ATP was calculated according to the standard curve of ATP.

### Antibiotic Bactericidal Assays

Antibacterial assay was carried out as described previously (Peng et al., 2015). Overnight bacterial cultures were diluted 1:100 in fresh LB medium and cultured at 30°C to an OD600 of 1.0. Bacterial cells were collected by centrifugation at 8,000 rpm for 5 min. The samples were then washed with sterile saline three times; suspended in M9 minimal media containing 10 mM acetate, 1 mM MgSO_4_, and 100 μM CaCl_2_; and diluted to an OD600 of 0.2. Fe^3+^ or/and ceftazidime were added and incubated at 30°C and 200 rpm for 6 h. To determine bacterial count, 100 μl of cultures was obtained and then serially diluted. An aliquot of 10μl of each dilution was plated in TSB agar plates and incubated at 30°C for 22 h. The plates only with 20–200 colonies were counted, and the colony-forming unit per milliliter was calculated.

### Measurement of Membrane Potential

Bacterial membrane potential was measured by BacLight Bacterial Membrane Potential Kit (Invitrogen). In brief, bacteria were diluted to 10^6^ CFU/ml and stained with 10 μl of 3 mM DiOC_2_, followed by incubation for 30 min. Samples were analyzed using a FACSCalibur flow cytometer (Becton Dickinson, San Jose, CA, United States). The green/red fluorescence was detected with 488–530/610 nm. The membrane potential was determined and normalized as the intensity ratio of the red fluorescence and the green fluorescence. Relative proton motive force (PMF) was determined by test samples compared with control samples.

## Results

### ROS Is Reduced in LTB4-R_*CAZ*_

**FIGURE 1:**
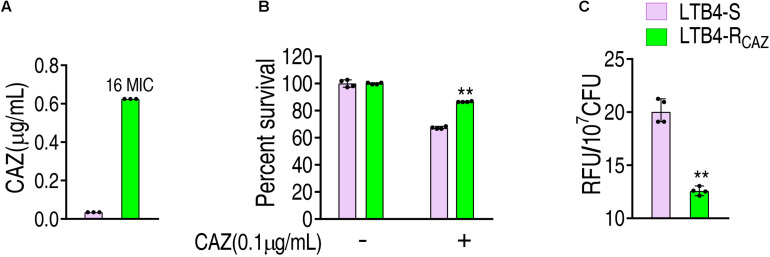
Ceftazidime resistance and reactive oxygen species (ROS) level in LTB4-R_*CAZ*_. **(A)** Minimum inhibitory concentration (MIC) of LTB4-S and LTB4-R_*CAZ*_. **(B)** Killing efficiency of ceftazidime to LTB4-S and LTB4-R_*CAZ*_. **(C)** ROS of LTB4-S and LTB4-R_*CAZ*_. Results **(B**,**C)** are displayed as mean ± SEM, as determined by two-tailed Student’s *t*-test. Four biological repeats are carried out. **P < 0.01.

LTB4 was cultured in LB medium with or without twofold serially diluted ceftazidime, leading to LTB4-R_*CAZ*_ and LTB4-S, respectively. The MIC of LTB4-S was 0.039 μg/ml CAZ, while that of LTB4-R_*CAZ*_ was 0.625 μg/ml CAZ. There was a 16-fold difference between the two strains (Figure 1A). Consistently, a higher viability was detected in LTB4-R_*CAZ*_ than LTB4-S (Figure 1B). To explore whether LTB4-R_*CAZ*_ had a reduced ROS production, ROS production was detected in LTB4-R_*CAZ*_ and LTB4-S. A lower ROS production was determined in LTB4-R_*CAZ*_ than LTB4-S (Figure 1C). These results indicate that ROS production is reduced in LTB4-R_*CAZ*_ compared with LTB4-S.

### ROS Promoter and Inhibitor Affect Ceftazidime Resistance and Ceftazidime-Mediated Killing

**FIGURE 2:**
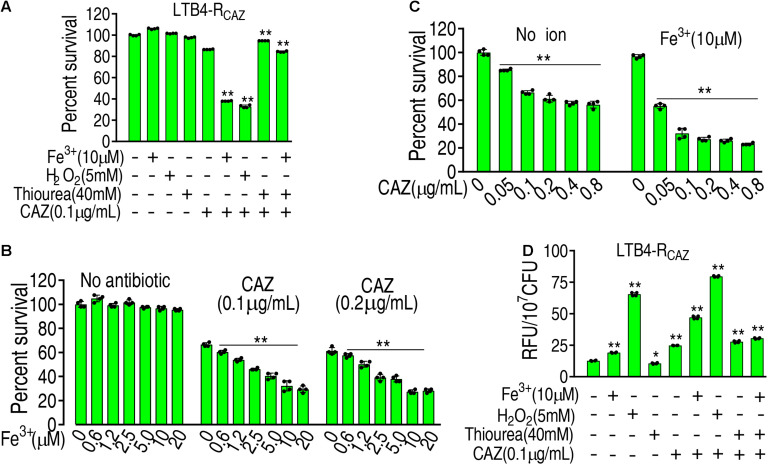
The role of ROS in ceftazidime-mediated killing. **(A)** ROS was quantified in LTB4-R_*CAZ*_ in the absence or presence of ceftazidime plus Fe^3+^, H_2_O_2_, or/and thiourea as indicated by fluorescence. **(B)** Percent survival of LTB4-R_*CAZ*_ in the presence of ceftazidime and the indicated concentrations of Fe^3+^. **(C)** Percent survival of LTB4-R_*CAZ*_ in the presence or absence of Fe^3+^ plus the indicated concentrations of ceftazidime. **(D)** ROS level in the presence or absence of ROS promoter and inhibitor plus ceftazidime. Results are displayed as mean ± SEM, as determined by two-tailed Student’s *t*-test. Four biological repeats are carried out. * < 0.05 and ** < 0.01.

These above results motivated us to speculate that ROS plays an important role in ceftazidime resistance. To demonstrate this, the viability of LTB4-R_*CAZ*_ was detected in the presence or absence of ROS promoter or/and inhibitor with ceftazidime. There was a stronger resistance to H_2_O_2_ in LTB4-S than *Escherichia coli* K12 and thereby 5 mM H_2_O_2_ was used (Supplementary Figure 1). The promoters Fe^3+^ and H_2_O_2_ (positive control) potentiated ceftazidime-mediated killing. However, the inhibitor thiourea not only eliminated the potentiation caused by the promoters but also inhibited the ceftazidime-mediated killing (Figure 2A). The effect of Fe^3+^ was increased in a dose-dependent manner and was related to the concentration of ceftazidime used (Figure 2B). When the concentration of Fe^3+^ was fixed, the killing efficacy was ceftazidime dose dependent (Figure 2C). To validate the role of the ROS promoter or/and inhibitor in the ceftazidime-mediated killing, we measured the ROS production of LTB4-R_*CAZ*_ in the presence or absence of the ROS promoter or/and inhibitor. ROS production was elevated and reduced in the presence of the inhibitor thiourea and promoters Fe^3+^, H_2_O_2_, or ceftazidime alone, respectively. Comparatively, the elevated ROS ranked as H_2_O_2_ > ceftazidime > Fe^3+^. When the synergistic use of ceftazidime with one of the promoters or/and the inhibitor was performed, they promoted and inhibited ROS level, respectively (Figure 2D). These results support the conclusion that ROS is related to ceftazidime resistance and promotes ceftazidime-mediated killing.

### The P Cycle Is Inactivated and the Activity of Antioxidant Enzymes Is Not Changed in LTB4-R_*CAZ*_

**FIGURE 3:**
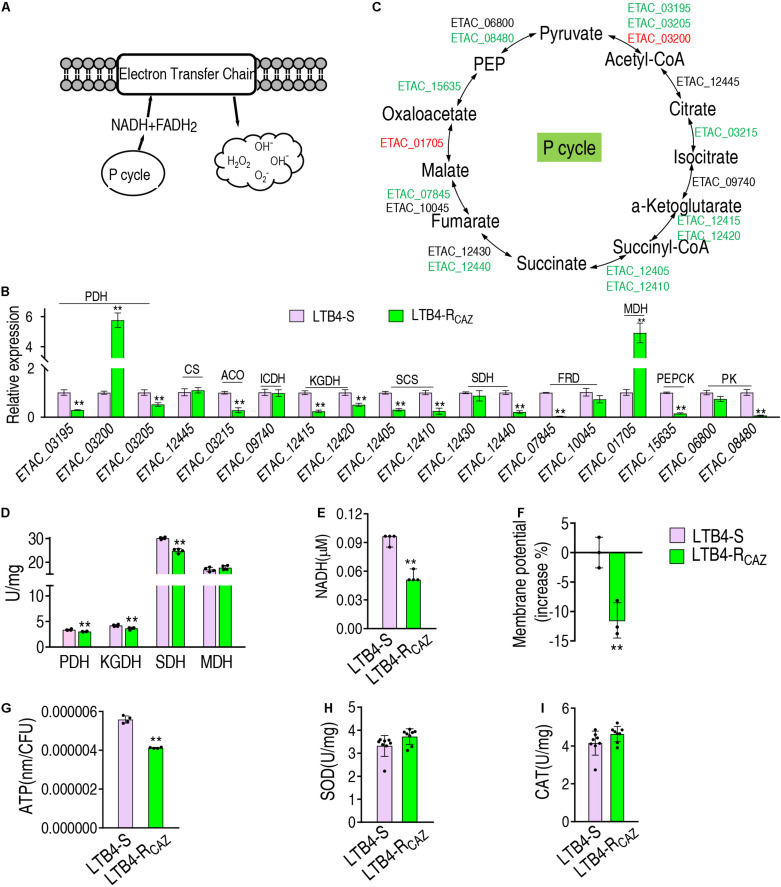
The P cycle, NADH, and ATP in LTB4-S and LTB4-R_*CAZ*_. **(A)** Sketch diagram describing the effect of the P cycle on ROS. **(B)** Real-time quantitative PCR (qRT-PCR) for the expression of the P cycle-related genes in LTB4-R_*CAZ*_ compared to LTB4-S. **(C)** A global view for transcription level of the P cycle genes. Red and blue indicate upregulated and downregulated expression, respectively. **(D)** The activity of PDH, KGDH, SDH, and MDH in the P cycle. NADH **(E)**, membrane potential **(F)**, and ATP **(G)** concentrations in LTB4-S and LTB4-R_*CAZ*_. The activity of superoxide dismutase (SOD) **(H)** and catalase (CAT) **(I)** in LTB4-S and LTB4-R_*CAZ*_. Results **(B,D–I)** are displayed as mean ± SEM, as determined by two-tailed Student’s *t*-test. Four biological repeats are carried out. **P < 0.01.

To understand why ROS are reduced in LTB4-R_*CAZ*_, the P cycle and SOD degradation were investigated. The P cycle is a recently illustrated cycle, which provides respiratory energy in *E. tarda* (Su et al., 2018), and is related to ROS biosynthesis (Figure 3A). For the investigation of the P cycle, qRT-PCR was used to measure the expression of genes in the P cycle. Among the 18 genes detected, 11, 2, and 5 were reduced, elevated, and unchanged, respectively. Specifically, the 11 reduced genes encode all enzymes detected except for citrate synthase (CS) and isocitrate dehydrogenase (ICDH). The two elevated genes encode PDH and MDH. The five unchanged genes encode CS, ICDH, SDH, fumarate reductase (FRD), and pyruvate kinase (PK) (Figures 3B,C). Consistently, a lower activity of PDH, KGDH, and SDH was detected in LTB4-R_*CAZ*_ than that in LTB4-S (Figure 3D). NADH, membrane potential, and ATP were reduced in LTB4-R_*CAZ*_ compared with LTB4-S (Figures 3E–G). These results indicate that the inactivation of the P cycle forms a characteristic feature in LTB4-R_*CAZ*_, which is related to the reduced ROS production. For the investigation of ROS degradation, the activity of ROS degradation enzymes was measured. Among these enzymes working for the ROS degradation, SOD and CAT are widely employed to indicate the antioxidant response. Therefore, the activity of SOD and CAT was detected in LTB4-R_*CAZ*_ and LTB4-S. A similar activity of SOD and CAT was measured between LTB4-R_*CAZ*_ and LTB4-S (Figures 3H,I). These results indicate that antioxidant response is not changed in LTB4-R_*CAZ*_. Taken together, the lower ROS in LTB4-R_*CAZ*_ is attributed to a reduction of ROS generation instead of an increase of ROS degradation.

### Fe^3+^ Impacts Metabolic Profile

**FIGURE 4:**
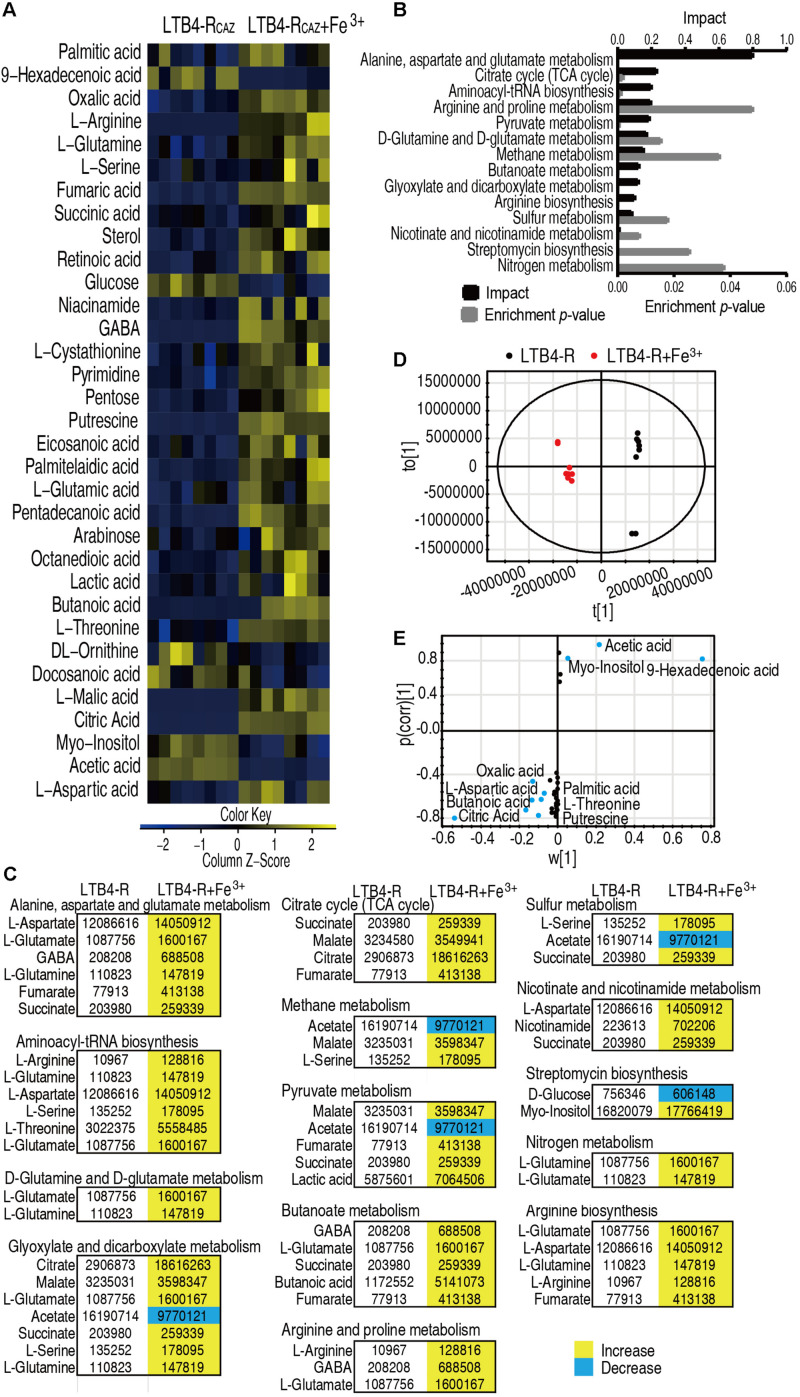
Differential metabolomics of LTB4-R_*CAZ*_ in response to Fe^3+^. **(A)** Heat map showing differential abundance of metabolites. Yellow and blue indicate increase and decrease of metabolites relative to the median metabolite level of the control, respectively (see color scale). **(B)** Pathway enrichment of varied metabolites in LTB4-R_*CAZ*_. **(C)** Integrative analysis of metabolites in significantly enriched pathways. Yellow and blue indicate increased and decreased metabolites, respectively. **(D)** PCA of LTB4-S and LTB4-R_*CAZ*_. Each dot represents the technical replicate analysis of samples in the plot. **(E)** S-plot generated from OPLS-DA. Predictive component *p* [1] and correlation *p* (corr) [1] differentiate LTB4-R_*CAZ*_ from LTB4-S. Dot represents metabolites, and candidate biomarkers are highlighted in blue.

The above results motivated us to explore whether Fe^3+^ promotes global metabolism including the P cycle to elevate ROS level and potentiate ceftazidime-mediated killing. To do this, GC-MS-based metabolomics was used to compare metabolic profiles between media with or without Fe^3+^. Four biological and two technical replicates were performed in each group, yielding eight data sets with 65 metabolites in a sample. Among the 65 metabolites, 33 (50.8%) showed differential abundance (*p* < 0.05), with 27 at higher abundance and 6 at lower abundance in the presence of Fe^3+^ (Figure 4A). Fourteen pathways were enriched, of which alanine, aspartate, and glutamate metabolism; the TCA cycle; and aminoacyl-tRNA biosynthesis were the top three pathways by impact (Figure 4B). Differential metabolites at abundance are listed in Figure 4C, where all detected metabolites were elevated in the TCA cycle (Figure 4C). Principal component analysis identified two principal components, where component t[1] distinguished LTB4-R_*CAZ*_ from LTB4-R_*CAZ*_ + Fe^3+^ (Figure 4D). Discriminating variables were identified on an S-plot (Figure 4E). Cutoff values were ≥ 0.05 for absolute value of the covariance *p* and ≥ 0.5 for correlation *p* (corr). Nine putative biomarkers were identified, including decreased abundance of 9-hexadecenoic acid, acetic acid, and myo-inositol and elevated abundance of citric acid, oxalic acid, putrescine, butanoic acid, threonine, aspartic acid, and palmitic acid. Among the elevated metabolites, citric acid plays a role in the P cycle. These results indicate that Fe^3+^ impacts the metabolic profile, where the P cycle is elevated.

### Fe^3+^ Activates the P Cycle

**FIGURE 5:**
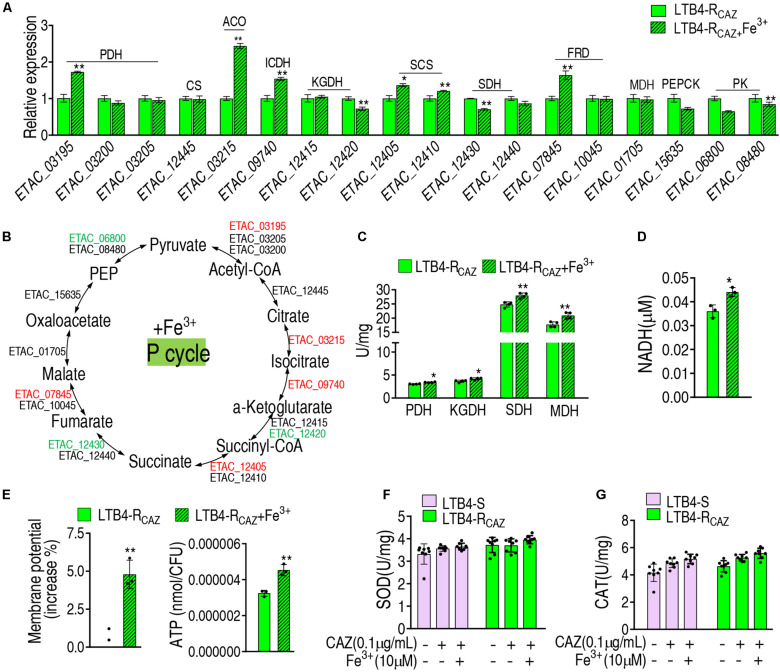
The effect of Fe^3+^ on the P cycle. **(A)** qRT-PCR for expression of the P cycle genes in the presence of Fe^3+^. **(B)** A global view for transcription level of the P cycle genes. Red and blue indicate upregulated and downregulated expression, respectively. The activity of enzymes of the P cycle **(C)**, NADH level **(D)**, membrane potential **(E)**, and ATP **(F)** of LTB4-R_*CAZ*_ in the presence or absence of Fe^3+^. **(G)** The activity of SOD and CAT in the presence or absence of ceftazidime or/and Fe^3+^. Results **(A,C–G)** are displayed as mean ± SEM, as determined by two-tailed Student’s *t*-test. Four biological repeats are carried out. *p < 0.05 and **p < 0.01.

To further demonstrate the Fe^3+^-mediated activation, gene expression and enzyme activity were measured in the P cycle. qRT-PCR analysis showed that out of 18 genes tested, 5, 3, and 10 were elevated, reduced, and unchanged, respectively. In detail, the five elevated genes encode PDH, ACO, ICDH, SCS, and FRD, and the reduced genes encode KGDH, SDH, and PK (these enzymes are encoded by two genes) (Figures 5A,B). The activity of PDH, KGDH, SDH, and MDH was elevated (Figure 5C), which was further supported by increased NADH, membrane potential, and ATP (Figures 5D–F). These results indicate that Fe^3+^ promotes the P cycle but does not affect the activity of SOD and CAT (Figure 5G).

### Fe^3+^ Promotes ROS via the P Cycle

**FIGURE 6:**
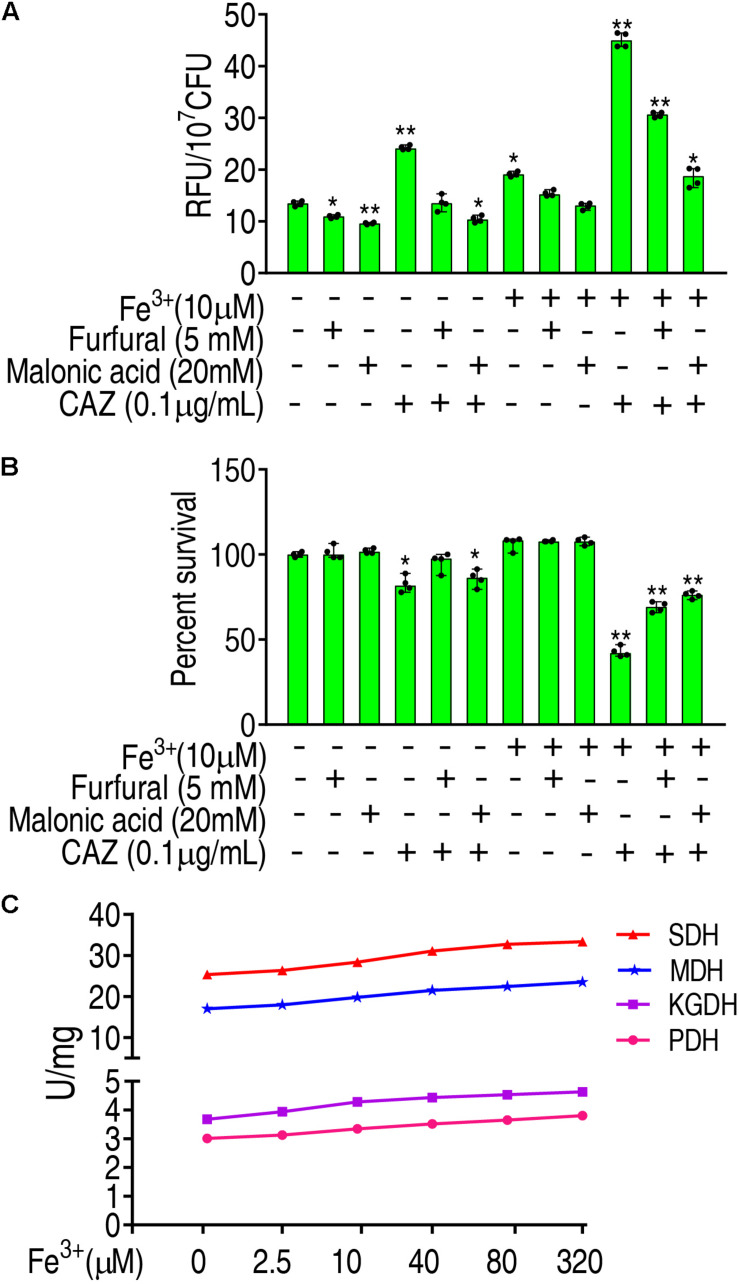
Promotion of Fe^3+^ to the P cycle. **(A)** ROS of LTB4-R_*CAZ*_ in the presence of Fe^3+^ and CAZ plus inhibitors of the P cycle. **(B)** Percent survival of LTB4-R_*CAZ*_ in the presence of Fe^3+^ and CAZ plus inhibitors of the P cycle. **(C)** The activity of PDH, KGDH, SDH, and MDH in the presence of Fe^3+^. Results are displayed as mean ± SEM, as determined by two-tailed Student’s *t*-test. Four biological repeats are carried out. *p < 0.05 and **p < 0.01.

To further validate that Fe^3+^ promotes ROS via the P cycle, inhibitors of the P cycle were used to investigate whether the inhibition affects ROS and viability of LTB4-R_*CAZ*_ in the presence of both Fe^3+^ and ceftazidime. Furfural is a non-competitive inhibitor for PDH, and malonic acid is competitive against SDH. Lower ROS were detected in the presence than the absence of furfural or malonic acid, while higher ROS were determined in the medium with than without Fe^3+^ in the presence or absence of ceftazidime and furfural or malonic acid (Figure 6A), suggesting that Fe^3+^ partly reverts the inhibition mediated by the two inhibitors. Consistently, a lower viability was detected in the medium with than without Fe^3+^ in the presence of furfural or malonic acid (Figure 6B). To understand the mechanisms by which Fe^3+^ promotes the P cycle, we supposed that Fe^3+^ activates the activity of enzymes in the P cycle. To explore this, the activity of PDH, KGDH, SDH, and MDH was measured in a medium with the indicated concentrations of Fe^3+^. The activity of the four enzymes was increased in a Fe^3+^ dose-dependent manner *in vitro* (Figure 6C). These results indicate that Fe^3+^ promotes the activity of PDH, KGDH, SDH, and MDH in the P cycle directly, which is related to the elevation of ROS level in the presence of Fe^3+^.

### Fe^3+^ Promotes the Cephalosporin-Mediated Killing to Clinically Isolated Pathogens

**FIGURE 7:**
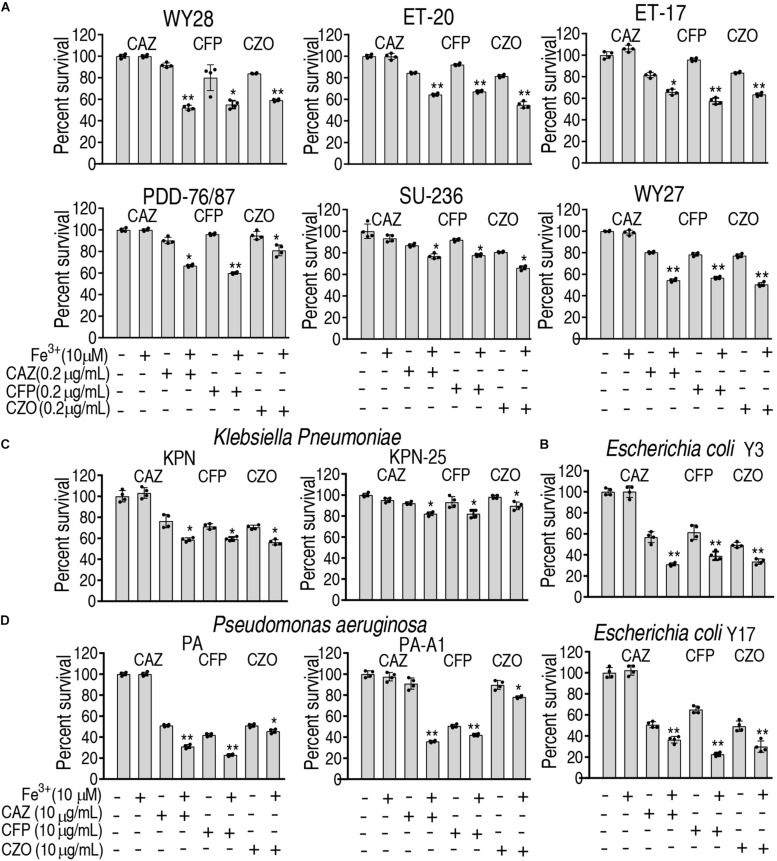
Percent survival of clinically isolated pathogens in the presence of Fe^3+^ and ceftazidime, cefoperazone, or cefazolin. **(A)** *E. tarda*. **(B)** *E. coli*. **(C)** *K. pneumoniae.* **(D)** *P. aeruginosa*.

To ensure the Fe^3+^-induced potentiation is generalizable to cephalosporins and clinically isolated pathogens, three types of cephalosporins (ceftazidime, cefoperazone, and cefazolin) and 12 strains of bacterial pathogens (*E. tarda*, *E. coli*, *Klebsiella pneumoniae*, and *Pseudomonas aeruginosa*) were used. The 12 strains of pathogens are resistant to at least three classes of antibiotics and thereby belong to multidrug-resistant bacteria. However, Fe^3+^ effectively promotes the three drugs to kill all pathogens (Figure 7). These results indicate that the Fe^3+^-induced potentiation is generalizable to the drugs and bacterial pathogens.

## Discussion

The present study first investigated whether ceftazidime resistance is related to ROS level and then explored whether Fe^3+^-potentiated ROS elevation in ceftazidime-mediated killing is related to the P cycle, which contributes to ROS biosynthesis. For this purpose, ROS level was compared between LTB4-S and LTB4-R_*CAZ*_. Lower ROS was detected in LTB4-R_*CAZ*_ than in LTB4-S. The reduction is accompanied with the inactivation of the P cycle. Since the P cycle is related to ROS generation, the inactivated P cycle should be one reason by which ROS is reduced in LTB4-R_*CAZ*_. Moreover, the role of ROS in ceftazidime-mediated killing was demonstrated by the ROS promoter Fe^3+^ and inhibitor thiourea. They increased and decreased the ceftazidime-mediated killing, respectively, suggesting that the ROS promoter and inhibitor affect the killing via regulation of ROS biosynthesis. These results indicate that the ceftazidime-mediated resistance and killing are regulated by ROS level. We further show that the Fe^3+^-induced potentiation is generalizable to ceftazidime and other cephalosporins to kill clinically multidrug-resistant pathogens *E. tarda*, *E. coli*, *K. pneumoniae*,and *P. aeruginosa*.

It has been reported that ROS level is a characteristic feature as a consequence of antibiotic resistance and sensitivity (Ma et al., 2016; Ye et al., 2018). Rosato et al. indicated that TCA cycle-mediated generation of ROS is a key mediator for MRSA survival under beta-lactam antibiotic exposure (Rosato et al., 2014). Hayakawa et al. (2019) demonstrated that anti-ROS agent prevents the acquisition of multi-drug resistance in clinical isolates of *P. aeruginosa*. Thomas et al. (2013) showed that a dysfunctional TCA cycle enables *Staphylococcus epidermidis* to resist oxidative stress and alter its cell surface properties, making it less susceptible to beta-lactam antibiotics. In addition, Battán et al. (2004) found that resistance to oxidative stress caused by ceftazidime and piperacillin in a biofilm of *Pseudomonas* is related to bacterial strains. On the other hand, ROS act as an antibiotic sensitizer for the treatment of antibiotic-resistant bacteria involved in infectious diseases (Guo et al., 2018). Recent observations have linked ROS production with bactericidal action of antibiotics, pointing to antibiotic-induced TCA cycle- and respiratory chain-dependent ROS production as playing a role in cell death (Dwyer et al., 2009; Van Acker and Coenye, 2017; Ye et al., 2018). The present study identified that both actions play a role. Specifically, decreased ROS production is a characteristic feature in LTB4-R_*CAZ*_, and ceftazidime-induced ROS production is required for ceftazidime-mediated killing in LTB4-R_*CAZ*_. These results indicate that the decreased ROS production contributes to the insensitivity to ceftazidime, and thereby reduction of ROS production is a mechanism by which *E. tarda* resist to ceftazidime.

The present study further explored why ROS production is reduced in LTB4-R_*CAZ*_ based on the P cycle-mediated generation and ROS degradation. It is documented that the reduction of ROS production is attributed to the decreased biosynthesis caused by inactivation of the P cycle instead of the degradation. The understanding of the biosynthesis of ROS is especially important for promoting antibiotic-mediated killing since ROS production contributes to antibiotic resistance. Wang et al. (2014) showed that loss of sigma(s) rendered stationary-phase *E. coli* more sensitive to the bactericidal antibiotic gentamicin due to a weakened antioxidant defense. Our recent publication indicates that alanine enhances aminoglycoside-induced ROS production through promoting ROS biosynthesis pathways and repressing transcription of antioxidant-encoding genes (Ye et al., 2018). Thus, a synergistic use of antibiotics with ROS generation promoter or/and ROS degradation inhibitor will elevate the antibiotic-mediated killing. In addition, metabolites promoting hosts to eliminate bacterial pathogens have been reported (Jiang et al., 2019a, b, 2020b; Gong et al., 2020a, b; Yang et al., 2020), where ROS are involved (Sarr et al., 2018; Gong et al., 2020b). Thus, it is possible to select the promoters or/and inhibitors that simultaneously elevate the ROS level of both hosts and bacteria. This approach will make both the host’s anti-infective ability and antibiotic-mediated killing play a role, having the most effect on the elimination of bacterial pathogens via ROS-mediated pathways.

A line of evidence has indicated that Fe^3+^ reduces ROS via the Fenton system (Song et al., 2020). However, information regarding whether Fe^3+^ regulates ROS by other ways is absent in bacteria. The present study showed that Fe^3+^ reverts the inactivity of the P cycle, which elevates ROS biosynthesis. Further evidences include the elevated activity of PDH, KGDH, SDH, and MDH in the presence of Fe^3+^ *in vitro*, suggesting that Fe^3+^ is an activator of these enzymes. Therefore, Fe^3+^ as a ROS promoter plays a role in the P cycle and Fenton system, which is not reported before in bacteria.

**FIGURE 8:**
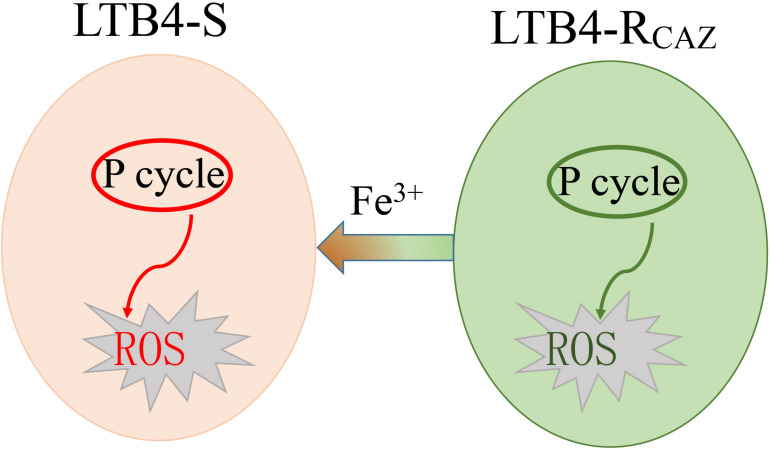
Sketch diagram describing a mechanism by which Fe^3+^ promotes ROS level.

In summary, ROS production is decreased as a crucial characteristic of LTB4-R_*CAZ*_, which is related to inactivation of the P cycle. Fe^3+^ promotes the activation of the P cycle and thereby elevates ROS level. The elevated ROS potentiates ceftazidime-mediated killing. When ROS inhibitor is used, the killing is reduced (Figure 8). These results expand our understanding for the role of Fe^3+^-induced ROS in antibiotic resistance. They may also provide tools and/or knowledge for future new strategies to stop infections by multidrug-resistant human pathogens.

## Data Availability Statement

The original contributions presented in the study are included in the article/Supplementary Material, further inquiries can be directed to the corresponding author/s.

## Author Contributions

HL conceived the study. JY and YS conducted the experiments. JY, YS, and HL analyzed the data. HL and XP wrote the manuscript. All authors contributed to the article and approved the submitted version.

## Conflict of Interest

The authors declare that the research was conducted in the absence of any commercial or financial relationships that could be construed as a potential conflict of interest.

## Footnotes

1
https://cran.r-project.org/web/packages/gplots/
