# Corrigendum: Reactive Oxygen Species-Related Ceftazidime Resistance Is Caused by the Pyruvate Cycle Perturbation and Reverted by Fe^3+^ in *Edwardsiella tarda*

**DOI:** 10.3389/fmicb.2021.778578

**Published:** 2021-10-06

**Authors:** Jinzhou Ye, Yubin Su, Xuanxian Peng, Hui Li

**Affiliations:** ^1^Center for Proteomics and Metabolomics, State Key Laboratory of Biocontrol, Southern Marine Science and Engineering Guangdong Laboratory (Zhuhai), School of Life Sciences, Sun Yat-sen University, Guangzhou, China; ^2^Key Laboratory of Functional Protein Research of Guangdong Higher Education Institutes, Department of Biotechnology, College of Life Science and Technology, Jinan University, Guangzhou, China; ^3^Laboratory for Marine Fisheries Science and Food Production Processes, Qingdao National Laboratory for Marine Science and Technology, Qingdao, China

**Keywords:** antibiotic resistance, reactive oxygen species, *Edwardsiella tarda*, the pyruvate cycle, ceftazidime

An author name was incorrectly spelled as “Jingzhou Ye.” The correct spelling is “Jinzhou Ye.”

The authors apologize for this error and state that this does not change the scientific conclusions of the article in any way. The original article has been updated.

## Publisher's Note

All claims expressed in this article are solely those of the authors and do not necessarily represent those of their affiliated organizations, or those of the publisher, the editors and the reviewers. Any product that may be evaluated in this article, or claim that may be made by its manufacturer, is not guaranteed or endorsed by the publisher.

