# Supplementary material for: Reactive Oxygen Species-Related Ceftazidime Resistance Is Caused by the Pyruvate Cycle Perturbation and Reverted by Fe3 + in Edwardsiella tarda
Source: Front Microbiol. 2021 Apr 28;12:654783. doi: 10.3389/fmicb.2021.654783 (PMC8113649; doi:10.3389/fmicb.2021.654783)
Supplement: Supplementary file 1 [file Presentation_1.pdf]

Supplementary figure and table

ROS-related Ceftazidime Resistance is Caused by the Pyruvate Cycle Perturbation and Reverted by Fe^{3+} in *Edwardsiella tarda*

Jingzhou Ye[1], Yubing Su[3], Xuanxian Peng[1,2], Hui Li[1,2] *

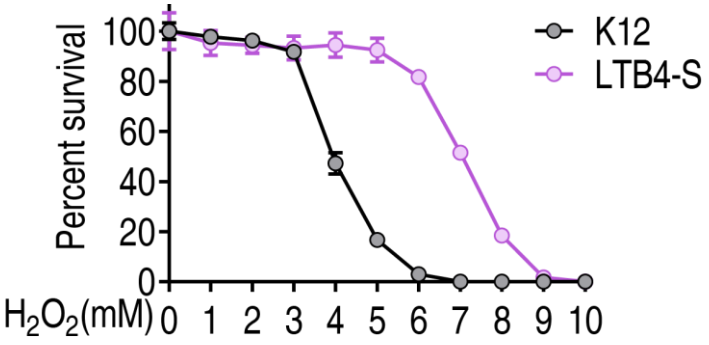

Supplementary figure 1**.** Resistance to H_2O_2 in LTB4-S and *E. coli* K12 at different concentration H_2O_2

SupplementaryTable 1 Primers used for qRT-PCR

KEGG entry	gene	Primer	Sequence (5'-3')
16SrRNA		Forward	GTAGTCCACGCTGTAAACGA
		Reverse	GAATTAAACCACATGCTCCA
ETAC_03195	*aceE*	Forward	GCGGTCACATAGCCTCCTATCA
		Reverse	AAGTTATTCAGCTGCTCCTCGGT
ETAC_03200	*aceF*	Forward	GCGTGAGTTTGGCGTTAACCT
		Reverse	TTGCTGAAGTCCACCTTCGG
ETAC_03205		Forward	GGGTAAGTTTACCTCGGCCAAC
		Reverse	CCCATCTCCAGGCCGATAAT
ETAC_12445	*gltA*	Forward	GATGTCTTCAAGAGCACCGTCAC
		Reverse	ATCTTGGAGAGCAGGCGGAA
ETAC_03215		Forward	CACTGTCCCATACTCTGCTGGTCT
		Reverse	TGGTTTCACCGCTGACCTTG
ETAC_09740		Forward	ATCTACACCGGAGAGAAGGCGA
		Reverse	CGCAGGCAGATATACAGATCCAG
ETAC_12415		Forward	GATGCTGACCACCTTTAACGAAGT
		Reverse	CCGCAATGCTGATATCGAAGTAG
ETAC_12420	*sucA*	Forward	ATCAGCAGGCCAACCTCGAT
		Reverse	TATTCTGCGCCGATGGTTCC
ETAC_12405		Forward	ACGCAGGACATGCTGGTGAT
		Reverse	TCTGCTTCACCGCCTCATAGGT
ETAC_12410	*sucC*	Forward	AGATTGCGCGCGAGCTGTAT
		Reverse	CAAACTGCTGGGCCTGCTTA
ETAC_12430	*sdhA*	Forward	CGTTTTCACGCCTGGAGGAT
		Reverse	GCATACCACTCGGAGAAGATCGT
ETAC_12440	*sdhC*	Forward	TTTGCCAACAATCCGCTTTC
		Reverse	ATCCCGCCGCAGATGTGATA
ETAC_07845		Forward	GAGAAGGCGGACGCCATTAT
		Reverse	CGTCATCATTGGGATGCACC
ETAC_10045		Forward	GACGGCGATTATCGTGGGTAA
		Reverse	ATTTGTACTCCTCGCCCTCGGT
ETAC_01705		Forward	GCGCGGATATCGTGCTGATT
		Reverse	TTCAGCACCTCAGCCGCAAT
ETAC_15635		Forward	TCTCCAACAAGCGCCTGTTC
		Reverse	GAAGTGATACTGCGTCTGATCCG
ETAC_06800		Forward	GACAAGGGCGAAGGCGATAA
		Reverse	TTTATTGTTGGACAGCGGGC
ETAC_08480		Forward	CCTGATCGGCATGGAAGTGA
